# The relationships between mild cognitive impairment and phenotype in Parkinson’s disease

**DOI:** 10.1038/npjparkd.2015.15

**Published:** 2015-08-27

**Authors:** Jennifer YY Szeto, Claire O’Callaghan, James M Shine, Courtney C Walton, Loren Mowszowski, Sharon L Naismith, Glenda M Halliday, Simon JG Lewis

**Affiliations:** 1 Parkinson’s Disease Research Clinic, Brain and Mind Research Institute, University of Sydney, Sydney, NSW, Australia; 2 Neuroscience Research Australia and the University of New South Wales, Sydney, NSW, Australia; 3 Healthy Brain Ageing Program, School of Psychology, University of Sydney, Sydney, NSW, Australia

## Abstract

**Background:**

The concept of differing clinical phenotypes within Parkinson’s disease (PD) is well represented in the literature. However, there is no consensus as to whether any particular disease phenotype is associated with an increased risk of mild cognitive impairment (MCI) using the newly proposed Movement Disorders Society diagnostic criteria for this feature.

**AIMS:**

To explore the expression of PD-MCI in relation to the heterogeneity of idiopathic PD.

**Methods:**

A cluster analysis incorporating a range of specific demographic, clinical and cognitive variables was performed on 209 patients in the early stages of PD (between Hoehn and Yahr stages I–III). *Post hoc* analyses exploring variables not included in the clustering solution were performed to interrogate the veracity of the subgroups generated.

**Results:**

This study identified four distinct PD cohorts: a younger disease-onset subgroup, a tremor dominant subgroup, a non-tremor dominant subgroup, and a subgroup with rapid disease progression. The present study identified a differential expression of PD-MCI across these subgroups, with the highest frequency observed in the non-tremor dominant cluster. The non-tremor dominant subgroup was also associated with a higher prevalence of freezing of gait, hallucinations, daytime somnolence, and rapid eye movement sleep behavior disorder compared with other subgroups.

**Conclusions:**

This study confirms the existence of heterogeneity within the early clinical stages of PD and for the first time highlights the differential expression of PD-MCI using the newly defined diagnostic criteria for this feature. An improved understanding of PD-MCI and its clinical relationships may lead to an improved understanding of the pathophysiology underlying heterogeneity in PD.

## Introduction

Parkinson’s disease (PD) is a progressive neurodegenerative disorder with a wide variety of clinical symptoms. In addition to classic motor features (i.e., tremor, rigidity, bradykinesia, and postural instability), non-motor symptoms are now widely accepted as part of the clinical spectrum. However, the specific pathophysiological processes underlying these features are not all well understood.

Severe dopamine depletion in the striatum due to loss of dopaminergic neurons in the nigrostriatal pathway is well recognized as the predominant histological feature of PD, regardless of the clinical phenotype. Dopamine has a key role in the regulation of corticostriatal pathways and functional connectivity is clearly important across a range of physical, cognitive, limbic, and other processes.^1^ Thus the loss of this neurotransmitter in PD is likely to account for many of the observed symptoms in PD. However in addition to nigrostriatal loss, Lewy pathology and neuronal loss across differing populations including cholinergic, serotonergic, and noradrenergic structures are also recognized as contributing factors to several motor (e.g., gait and balance) and non-motor symptoms (e.g., cognitive and affective disturbances) that are experienced by PD patients.^2^ These pathologies lead to neurochemical deficits, thereby disrupting the modulation of neuronal signaling across cortical and subcortical regions crucial for normal motor and non-motor functioning.^3^

### Mild cognitive impairment in PD

The identification of mild cognitive impairment (MCI) has been increasingly recognized as a transitional state between normal aging and dementia in other patient populations (e.g., Alzheimer’s disease). In PD, MCI is a common manifestation that can be present even at diagnosis and represents a significant risk factor for the development of dementia (PDD).^4^

To facilitate early identification of the distinct PD cohort at risk of developing PDD, a definition of MCI in PD (PD-MCI) has recently been formulated. The definition of PD-MCI describes patients with cognitive deficits who have preserved capacity for daily functioning and do not fulfill the criteria for PDD.^5^ It is anticipated that in future these criteria may be used to identify those patients who would benefit most from any potential dementia intervention strategies.

### PD-MCI and heterogeneity in PD

Although the existence of PD-MCI is widely recognized, the clinical features associated with it have yet to be well characterized. The concept of distinct clinical phenotypes in PD is well represented in the literature.^6–11^ Certain characteristics, such as age of disease onset and predominant motor subtypes, have been most widely used to identify distinct PD cohorts, as well as their association with cognitive impairment. In general, these studies indicate that PD patients whose motor dysfunction is principally characterized by non-tremor features have more severe cognitive impairments than those with tremor dominant symptoms. The effect of age of onset on cognitive abilities in PD patients is more controversial. Some studies have shown that patients with a younger age of PD onset have more preserved cognitive performance compared with those with older age of onset despite being of equivalent age.^12^ However, other work has reported that early onset PD patients may have more severe cognitive impairments.^13^

Many of the previous studies exploring heterogeneity in PD have utilized the classification of distinct cohorts based on an arbitrary division of patients into prospectively defined subgroups, which may lead to an inherent bias in their conclusions. To avoid such limitations, data-driven analysis, which seeks to divide patients into discrete clusters based on a number of disease features that are assessed in conjunction with each other, can help to minimize this bias. In general, previous studies based on the data-driven approach have observed variable degrees of cognitive impairment across the subgroups. However, none of these existing studies were able to evaluate the presence of PD-MCI as defined by the new diagnostic Movement Disorder Society (MDS) criteria.^5^

As with cognitive impairment, various PD phenotypes may also be associated with different risks and severities of other motor and non-motor symptoms that are commonly comorbid (e.g., depression and sleep disturbances).^14^ The incidence of such symptoms may aggravate pre-existing cognitive deficits, thereby increasing the risk of MCI in a specific PD subgroup. Therefore, it is important that such distinct relationships, if they exist, be explored as they may reflect differing patterns of underlying pathological change. Previous clinicopathological research has investigated the progression of pathology in PD subgroups and observed differences in Lewy pathology and an association with non-motor symptoms such as dementia and depression.^8^

In light of these issues, the current study aimed to explore the differential expression of MCI and associated clinical symptoms across PD subgroups derived using a data-driven cluster analysis approach. This study focused on PD patients in the earlier clinical stages given that clinical symptoms in patients with more advanced PD can often be confounded by co-existent pathologies. A greater understanding of how PD-MCI relates to heterogeneity in PD would likely have implications on future studies regarding the etiology of this emerging entity and for future neuroprotective approaches targeting PDD. The identification of subgroups of patients who are characterized by interrelated features of cognitive impairment and other clinical symptoms may also offer the prospect of more coordinated management addressing patterns of disease rather than individual symptoms.

## Materials and methods

### Participants

This study included a total of 209 participants with PD between Hoehn and Yahr stages (H & Y) I and III (144 men, 65 women; mean age=66.68 (s.d.=8.90); mean disease duration=5.87 (4.92) years) from the Parkinson’s Disease Research Clinic at the Brain and Mind Research Institute, University of Sydney. The diagnosis of idiopathic PD was based on the United Kingdom Parkinson’s Disease Society Brain Bank criteria, and was confirmed by a neurologist (SJGL). Participants meeting MDS criteria for PDD were excluded.^15^ Only participants with complete datasets were included in the analysis. The research was approved by the Human Research Ethics Committee of the University of Sydney, and written informed consent was obtained from all participants.

### Clinical assessment

All participants underwent neurological and neuropsychological assessment as previously reported elsewhere.^16^ Patients were tested whilst on their usual medications. The neurological evaluation rated participants according to H & Y stages, and assessed them on the revised MDS Task Force Unified Parkinson’s Disease Rating Scale (MDS-UPDRS). Details of age at disease onset, symptoms at onset, disease duration, and medications were also recorded. A detailed medication history was taken for descriptive purposes. Of the 209 participants, 186 were taking medications for PD: 74 were treated with L-dopa monotherapy (one of whom also had electroconvulsive therapy), whereas further 71 were taking L-dopa with adjunctive medications (four of whom also had deep-brain stimulation) including dopamine agonists (50 participants), monoamine oxidase inhibitors (10 participants), catechol-*O*-methyl transferase inhibitors (7 participants), antiparkinson agents (1 participant), and anticholinergics (3 participants). Thirty-one participants taking L-dopa plus adjunctive dopamine agonists were also taking catechol-*O*-methyl transferase inhibitors, monoamine oxidase inhibitors, antiparkinson agents, or benzodiazepine. Ten participants were treated with dopamine agonist monotherapy. Of the 209 participants, 26 were also taking antidepressant medications. Twenty-three participants were untreated.

### Neuropsychological assessment

All patients underwent a neuropsychological battery comprising the following tests: the revised National Adult Reading Test (an estimate of premorbid intellectual functioning), Mini-Mental State Examination (a measure of global cognitive function), Trail Making Test A (total time; attention/visuomotor processing speed), Trail Making Test B (total time; executive functioning), digit span subtest from the Wechsler Adult Intelligence Scale-III (total score; working memory), Logical Memory I (total score; memory), Logical Memory II (delayed recall total; memory), Controlled Oral Word Association Test phonemic fluency (letters: FAS; executive functioning), and Controlled Oral Word Association Test semantic fluency (animals: total score; language).

### Diagnosis of PD-MCI

A diagnosis of PD-MCI was made according to the recently proposed MDS Task Force Level 1 criteria.^5^ PD-MCI was diagnosed when: (1) impairment was observed on two or more neuropsychological tests, defined by at least 1.5 s.d. below premorbid level of cognitive functioning, and (2) subjective cognitive problems were reported by patients or family members as defined by a score of 1 or more on item 1 (cognitive impairment) of the MDS-UPDRS part I. Sub-classification of PD-MCI was not performed as this may only be completed when utilizing a Level 2 criteria neuropsychological battery.

### Self-report measures

All participants completed the Parkinson’s Disease Questionnaire (PDQ-39), which measures health-related quality of life. This questionnaire consists of 39 items covering the following eight dimensions: mobility, activities of daily living, emotional wellbeing, stigma, social support, cognition, communication, and bodily discomfort. Scaled scores on the PDQ-39 range from 0 to 100, with higher scores indicating poorer quality of life. Severity of depressive symptoms was derived from the 21-item Beck Depression Inventory-II, and a cutoff score 13/14 was used to determine (at least moderately) significant symptoms. The Epworth Sleepiness Scale was used to measure daytime sleepiness. Scores are scaled between 0 and 24, with a higher score indicating higher levels of daytime sleepiness. A cutoff score of 10 was used to determine patients with excessive daytime sleepiness.^17^ The Scales for Outcomes in PD-Sleep Scale (SCOPA-S) was used to evaluate both daytime and night-time sleep disturbances. Scores range from 0 to 18 for daytime sleepiness and 0 to 15 for night-time sleep quality. Cutoff scores of 4/5 and 6/7 for the SCOPA-S (DS) and SCOPA-S (NS) were used respectively to determine the presence of sleep disturbances.^18^ To identify patients with rapid eye movement (REM) sleep behavior disorder (RBD), the REM Sleep Behavior Disorder Screening Questionnaire was used. Scores range between 0 and 13 (higher scores indicate more severe symptoms), and a cutoff score of 5 was used.^19^ The Freezing of Gait Questionnaire (FOG-Q) item 3 (‘Do you feel that your feet get glued to the floor while walking, making a turn or when trying to initiate walking (freezing)?’) was used to identify patients with freezing. This measure has previously been shown to be a reliable screening tool for identifying ‘freezers’, with a positive score indicating ‘freezers’. The Scales for Outcomes in Parkinson’s disease-Psychiatric Complications (SCOPA-PC) item 1 was used to identify patients who hallucinate and confirmed by a score of 1 or more on item 2 (hallucinations and psychosis) of the MDS-UPDRS part I.

### Derivation of variables included in the cluster analysis

Variables included in the cluster analysis comprised standardized values for motor phenotype score, age of disease onset, rate of disease progression, revised National Adult Reading Test, Mini-Mental State Examination, Logical Memory II, Trail Making Test B, Beck Depression Inventory-II and dopaminergic therapy. These variables were selected from a broad range of phenotypic features that have been highlighted in previous studies as being significant in disease heterogeneity.^6,7^

Rate of disease progression was derived from dividing the total MDS-UPDRS score for sections I–III by the disease duration (years), in order to allow comparison between patients with differing disease durations assessed at only a single time point.^6^ We also compared tremor and non-tremor symptoms with the motor phenotype score.^6^ The score was obtained by dividing a patients’ tremor score by their non-tremor score. Aligning with the approach used in previous research, only items with the higher lateralized score (left or right) were selected. The tremor score represents the severity of subjective tremor and objective tremor at rest and during movements. This score consisted of the mean of items 23, 54 or 55 (depending on the highest lateralized score), and 59. The non-tremor score assesses speech, facial expression, swallowing, ability to turn in bed, postural stability, walking and posture, rigidity, and global spontaneity of movement. This score was derived from the mean of items 28–44 (only items with the highest lateralized score) of the MDS-UPDRS. Impairment on the Trail Making Test B and Logical Memory II was defined as 1.5 s.d. below normative data. In keeping with previous work, dopaminergic therapy was calculated as an ordinal variable in the cluster analysis, with a pre-defined scale ranging from 0 to 2.^6^ A score of 0 represented no treatment with L-dopa or dopamine agonist (DA). A score of one was given to patients taking below 1,000 mg of L-dopa per day with/without a DA, or DA monotherapy. A score of two represented L-dopa dose over 1,000 mg with/without concomitant DA.

### Statistical analyses

Non-hierarchical (*k*-means) cluster analyses were performed between 2- and 5-cluster solutions, which have previously been considered clinically useful in typing the spectrum of PD patients.^7^ The optimal number of clusters was determined by local peaks of the cubic cluster criterion. Subsequent *post hoc* analyses were undertaken to compare the patient subgroups. The Kolmogorov–Smirnov test was performed to determine normal distribution. One-way analysis of variance followed by unpaired *t*-tests, or the Kruskal–Wallis test followed by Mann–Whitney *U*- tests (depending on whether the variable met parametric assumptions) were used to validate and further specify the obtained cluster solution. *Post hoc* analyses were also performed to validate the solution found in the exploratory cluster analysis. This included related independent variables not included in the cluster analysis: age, disease duration, H & Y scale and the cognitive impairment dimension of the PDQ-39. All analyses employed an alpha level of *P*⩽0.05, and were two tailed.

## Results

### Explorative cluster analysis

Using cubic cluster criterion, a four-cluster model was selected as the most relevant: (1) a subgroup of patients with a younger age of disease onset (YO), (2) a subgroup of tremor dominant (TD) patients, (3) a subgroup of non-tremor dominant (NTD) patients, and (4) rapid disease progression (RDP) subgroup. The characteristics of the identified clusters are summarized in Table 1.

The frequency of significant depressive symptoms in this sample was 27%. Cluster 1 comprised 45% of the sample. Patients in this cluster were characterized by a younger disease onset, compared to patients in the other three clusters. The second patient profile (cluster 2) comprised 12% of the sample. Patients in this cluster were characterized by having a tremor dominant motor phenotype score, lower L-dopa dose, and no statistically significant cognitive impairments. Cluster 3 comprised 23% of the sample. Patients in this cluster were characterized by having a non-tremor dominant motor phenotype score. These patients showed cognitive impairment most clearly demonstrated by a significantly lower Trail Making B score and Mini-Mental State Examination score, as well as a higher L-dopa dosage. Cluster 4 comprised 21% of the sample. Patients in this subgroup demonstrated a rapid course of disease progression but no severe cognitive impairment or motor disability compared with the other subgroups (Table 1). The L-dopa dose of these patients was similar to the doses taken by the tremor dominant cohort, but was significantly less than those that of the younger onset cohort, and the non-tremor dominant cohort.

### Validation of the cluster solution

Demographic and clinical variables including age, disease duration, H & Y score, and PDQ-39 score were used to confirm the cluster solution determined by the explorative *k*-mean cluster analysis (see Table 2). A comparison of mean age at assessment among the four clusters showed significant differences. Pairwise cluster comparisons indicated that YO patients had a significantly lower mean age at assessment than those from the other clusters—TD, NTD, and RDP, respectively (*t*=5.17, *P*<0.001; *t*=4.70, *P*=0.011; *t*=31.70, *P*<0.001).

Mean-disease duration scores also showed significant differences across the clusters. Patients from the RDP cluster had a significantly shorter duration of disease compared with those from the YO, TD, and NTD clusters, respectively (*U*=558.00, *z*=−6.86, *P*<0.001; *U*=375.50, *z*=−1.96, *P*=0.05; *U*=169.00, *z*=−6.93, *P*<0.001).

A comparison of mean scores on the H & Y showed significant differences among the four clusters. Patients from the TD cluster had a significantly lower H & Y score than those from YO, NTD, and RDP clusters, respectively (*U*=685.50, *z*=−3.17, *P*=0.002; *U*=260.00, *z*=−4.03, *P*<0.001; *U*=308.50, *z*=−3.04, *P*=0.002).

Results on the PDQ-39 questionnaire also added validity to the four-cluster solution, with patients from the four-cluster solution demonstrating significant differences in self-reported cognitive impairment. Patients from the NTD cluster had higher scores on the ratings for cognitive impairment than those from the YO, TD, and RDP clusters, respectively (*U*=1512.50, *z*=−3.15, *P*=0.002; *U*=297.00, *z*=−1.95, *P*=0.50; *U*=617.50, *z*=−3.18, *P*=0.001).

### Frequency of PD-MCI

Substantial differences in the frequency of PD-MCI were observed in different clusters. Patients from the NTD subgroup had the highest frequency, with 26 out of 48 patients (54%) characterized as having PD-MCI. This was followed by the TD subgroup, which had 10 out of 24 patients (42%) diagnosed as PD-MCI. The frequency of PD-MCI in the YO and RDP subgroups was 24% (22 out of 93 patients) and 25% (11 out of 44 patients), respectively.

### Other clinical symptoms

Table 3 shows the percentage of patients in each cluster who scored above the cutoff to indicate clinically significant symptoms on the SCOPA-S (NS), SCOPA-S (DS), Epworth Sleepiness Scale, REM Sleep Behavior Disorder Screening Questionnaire, or who were categorized as manifesting symptomatology on the basis of a positive answer to FOG-Q item 3, and SCOPA-PC item 1.

Substantial differences in frequencies were observed between the NTD subgroup and the other subgroups on FOG-Q item 3, SCOPA-PC item 1, SCOPA-S (DS), SCOPA-S (NS), Epworth Sleepiness Scale, and REM Sleep Behavior Disorder Screening Questionnaire, with the NTD subgroup having the highest percentages in these measures amongst the four subgroups (see Table 3).

## Discussion

Using a data-driven approach, this study is the first to report the frequency of MCI across PD subgroups using the newly proposed MDS diagnostic criteria. Consistent with previous findings,^6,7^ we identified four distinct PD cohorts: (1) a younger disease-onset subgroup, (2) a tremor dominant subgroup, (3) a non-tremor dominant subgroup, and (4) a subgroup with rapid disease progression. In addition to identifying the prevalence of PD-MCI across these four subgroups, this study explored their associated clinical relationships. Importantly, we demonstrated that the four PD subgroups were associated with a differential expression of PD-MCI with the highest PD-MCI frequency observed in the NTD phenotype. This subgroup was also associated with the highest prevalence of FOG, hallucinations, daytime somnolence and RBD than the other subgroups.

Heterogeneity in PD with respect to predominant motor phenotype is well represented in the literature.^6–10^ In support of previous work, the NTD subgroup identified here exhibited greater cognitive impairment compared with other subgroups.^6,10^ Most importantly, a higher prevalence of PD-MCI was observed in this subgroup affecting over half of the patients (54%). Such differences in PD-MCI frequencies may reflect more profound neuropathological lesions in patients with NTD symptoms, in particular with regards to nigral pathology.^20^ Evidence from *in vivo* dopaminergic imaging has confirmed more severe nigral pathology in patients with NTD features, relative to TD patients.^21^ Given that dopamine has a key role in the regulation of corticostriatal as well as limbic (mesolimbic) and cortical (mesocortical) pathways, more profound dopamine dysfunction is also likely to have greater impact upon the functional connection between regions involved in cognitive processes.^1^ Taken together, the interplay between deficits in different dopaminergic systems may at least in part relate to the disease heterogeneity observed in the present study.

In addition, the NTD subgroup was the only subgroup to demonstrate deficits on the Trail Making Test B. This may indicate more selective cognitive dysfunction (i.e., executive dysfunction) that was not present in the other subgroups. The presence of selective cognitive dysfunction in the NTD subgroup may reflect greater dysfunction within the frontostriatal circuitry. However, whether this select deficit in the NTD subgroup would confer an increased risk for developing PDD remains to be proven. Indeed, whereas previous research has reported that posterior-cortically based cognitive deficits have a high likelihood of transitioning to dementia, this may not be the case for selective executive dysfunction.^22,23^ Longitudinal follow-up of NTD cohorts would therefore be an important future direction, in order to establish rates of transition to dementia in this subgroup, who appear to manifest the highest prevalence of MCI.

Beyond the influences of dopaminergic pathology, it has become increasingly apparent that PD is better described as a multi-system neurodegenerative process that involves an interplay between a host of non-dopaminergic pathways, including noradrenergic, serotonergic, and cholinergic cell populations.^24,25^

Previous neuropathological and neuroimaging studies have suggested that degeneration of the cholinergic system may have a major role in the etiology of non-tremor motor features in PD including postural instability and gait.^2,24^ Involvement of the cholinergic system has also been discussed extensively in cognitive dysfunction and dementia in PD.^2,24,26^ Cortical acetylcholinesterase is a reliable marker of cholinergic pathways and depletion of this neurotransmitter system, as characterized by positron emission tomography, has been found to correlate with poorer executive and attentional functions, as well as more general cognitive impairment.^27^ In fact, a recent study reported that apart from more substantial dopaminergic deficits, PDD patients also exhibited additional cholinergic deficits compared with nondemented PD patients.^26^ Furthermore, adverse effects on attentional and executive processes have been observed following the administration of anticholinergic drugs in PD patients with mild cognitive symptoms.^28,29^ Considering the role of altered cholinergic neurotransmission on motor and cognitive functioning in PD, the higher PD-MCI prevalence in the NTD subgroup may further reflect a more widespread pathology that includes additional cholinergic denervation compared with other subgroups. However, it remains to be determined whether such deficits act in isolation on discrete cognitive functions or sequentially with nigral pathology.^26^

In addition to the cognitive deficits identified, results from the present study demonstrated that patients in the NTD subgroup also had a selective pattern of other motor and non-motor symptoms including FOG, hallucinations, daytime somnolence, and RBD as compared with the other subgroups. Finding increased rates of FOG in the NTD patient subgroup is consistent with previous work, which has demonstrated that PD patients with initial NTD symptoms (i.e., bradykinesia and rigidity) rather than those with TD symptoms were more likely to develop FOG.^30^ At present the pathophysiology underlying FOG is not well understood, although lesions of the dopaminergic and cholinergic systems are thought to be critically involved.^31^

NTD patients were also more likely to experience hallucinatory symptoms.^6,7^ Hallucinations in PD have traditionally been considered as a dopamine-related phenomenon, with the first line of management often involving a reduction in dopaminergic medication. However, it is now apparent that the development of hallucinations in PD patients may arise from the complex neuropathology of advancing disease. A clinicopathological correlation between well-formed visual hallucinations and high densities of Lewy bodies in the amygdala and parahippocampus has been described in previous research.^32^ Furthermore, a wide range of neurotransmitter systems have also been implicated in the pathogenesis of hallucinations in PD. For instance, cholinersterase inhibitors, such as Rivastigmine, have been documented as improving hallucinatory symptoms in PD patients.^33^ Furthermore, medications targeting other neurotransmitter systems, such as Clozapine, an atypical antipsychotic with both antidopaminergic and antiserotonergic properties as well as Pimavanserin, a serotonin 2A receptor inverse agonist, have also been shown to improve PD-related psychosis.^34,35^

The NTD subgroup was found to be associated with higher prevalence of daytime somnolence and RBD than the other subgroups. Although the higher prevalence of daytime somnolence and RBD in the NTD subgroup may be associated with their higher L-dopa doses, which is a well-documented contributing factor of sleep disturbances in PD,^36,37^ these symptoms may also arise from disrupted neurobiological changes in the ascending arousal system.^38^ Thus, it is likely that the pathophysiology of these additional non-motor symptoms is related to a range of neural pathways that are more affected in the NTD subgroup than other subgroups.

Previous studies have shown strong links between cognitive impairment, FOG, hallucinations, daytime somnolence, and RBD in PD patients.^39–41^ The incidence of these features and PD-MCI in the NTD subgroup and the number of previous studies associating these features with the involvement of multiple but similar neural pathways suggests common or parallel underlying pathological processes. Alternatively, these features could arise secondary to, or aggravated by other pathologies such as Alzheimer’s disease.^42,43^ Indeed the frequency of comorbid pathology at post-mortem is significant^44^ but what is less well understood, is how such changes might impact on the potential phenotypes. Similarly, whilst there is some data highlighting the influence of genetic polymorphisms on cognitive decline in PD, such as the α-Syn gene (SNCA)^45^ and the catechol-*O*-methyl transferase gene,^46^ little is known on how these might affect other phenotypic features. These findings have important implications for further research regarding the differing underlying pathophysiology in different subtypes and their association with PD-MCI.

The RDP subgroup identified in this study matches a similar profile of one that has been identified previously by a number of independent research teams.^6–8^ The cluster analysis in the present study produced a RDP subgroup that accounted for 21% of the total sample, which falls within the range of previous studies.^6,7,9,11^ Again, RDP was associated with older than average age of disease onset, intact cognitive functioning, and the taking of relatively small doses of dopaminergic therapy. The association between an older age at disease onset and a rapid course of disease progression may be due to less compensatory function or brain reserve and potentially less effective immunity to PD pathology. Selikhova and colleagues^8^ carried out a clinicopathological study of PD subgroups based on the data-driven subtype classification proposed by Lewis *et al.*^6^ They found that unlike the other three groups, non-Lewy body pathology (e.g., Alzheimer’s) and dementia was not present in the RPD subgroup, and this subgroup had significantly lower and more restricted Lewy body pathology than the other subgroups. The current study extended previous observations by confirming that this group was linked to a relatively lower prevalence of PD-MCI, FOG, hallucinations, daytime somnolence, and RBD. It remains unclear what contributes most to these clinical differences in the RDP subgroup. It is possible that this group harbors some cases of well-known PD mimics, which can be very difficult to distinguish clinically such as Multiple System Atrophy.^47^ Future research exploring the longitudinal progression and neuropathological basis for the rapid progression of this subgroup will be necessary.

The majority of patients (45%) in the present study were classified as being of YO, which is similar to rates reported elsewhere 29–61%.^6,7,9,11^ This proportion may be artificially inflated by the greater likelihood that such patients will more actively volunteer to participate in research studies. The finding that the YO subgroup had more preserved cognitive function is also aligned with previous reports.^6,7^ In addition, the current study extends this observation by demonstrating the relatively lower prevalence of PD-MCI (24%) in this phenotype compared with other subgroups. However, given that this subgroup were significantly younger than those in the other three subgroups, it remains to be investigated whether these patients are able to retain such clinical advantage as they age. Indeed, whilst relatively few patients with early onset PD in the Selikhova *et al.* ^8^ clinicopathological study developed dementia over the first 10–15 years of the disease, a substantial number of them eventually had developed features of advanced PD including cognitive disability as they aged. Similarly, longitudinal research has reported that most patients eventually develop PDD at ~70 years of age irrespective of the time of PD onset.^48^ On the contrary, evidence from genetic and epidemiological research showed that this was not the case in the majority of YO PD patients with PARKIN mutations, which are the most common cause of YO PD.^49^

### Limitations

This study employed Level 1 diagnostic criteria for PD-MCI, and therefore MCI subtyping (e.g., amnestic versus non-amnestic) was beyond the current scope of research conducted here. Exploring MCI subtypes as they manifest across different clinical phenotypes will be a key next step in understanding the interaction between heterogeneity and cognitive impairment in PD. However, whilst the employment of additional neuropsychological tests may increase characterization of PD-MCI subtype, the administration of a more comprehensive battery of neuropsychological tests may not always be practical or available due to the long administration time and the increased possibility of fatigue, particularly in this older population.^16^

Another limitation of this study relates to the relatively low prevalence of depression in the current sample (27%), as compared with that of ~40%^50^ shown in previous research. However, it is noted that we used Beck Depression Inventory-II cutoff scores for moderate symptomatology in this study, as opposed to a Diagnostic and Statistical Manual of Mental Disorders—Fourth Edition diagnosis and thus our findings are not directly comparable. In addition, future studies incorporating neuropathological investigations and exploration of predisposing genetic polymorphisms will also be critical in establishing the shared and unique pathological signatures of PD-MCI across the clinical phenotypes. This is particularly relevant as most neuropathological research to date has been focused on TD and NTD subgroups, so there are only scant data on the neuropathological correlates of YO and RDP subgroups. A prospective longitudinal clinicopathological follow-up of the cases studied here is being undertaken, which hopefully will eventually provide insight into the factors contributing to the neurobiology underpinning these distinct clinical subgroups.

In conclusion, using a data-driven approach this study confirmed the existence of heterogeneity within the early clinical stages of PD with four subgroups: (1) YO, (2) TD, (3) NTD, and (4) RDP; and importantly, this describes the differential expression of PD-MCI and other clinical symptoms across the identified subgroups. Nevertheless, rather than as a definitive classification system, the existence of these subgroups serves more as a platform for testing hypotheses. Avoiding the inclusion of key neuropsychiatric features such as hallucinations, as variables in the clustering solution was intended to allow the exploration of the subgroups generated without the significant influences of symptoms that would be regarded as less frequent in an early clinical disease sample. The confirmation of these subgroups may suggest the presence of different patterns of neuropathology that are likely to influence prognosis and response to treatment and provide an impetus for determining these clinicopathological correlations. Our current findings suggest that it is important to take into account the subgroup of individual patients when evaluating PD-MCI so as to better direct their specific management by taking into consideration the constellation of clinical symptoms that are likely to coexist and impact on quality of life.

## Figures and Tables

**Table 1:** Group characteristics for the four-cluster solution

	*YO*	*TD*	*NTD*	*RDP*	*Between-group differences*
Total number (%)	93 (45%)	24 (12%)	48 (23%)	44 (21%)	—
Age onset, years	56.63 (8.2)	62.28 (8.3)	59.86 (9.3)	69.87 (7.0)	*F*=26.22, df=3, *P*<**0.001**
Rate of disease progression, years	5.29 (3.1)	7.49 (6.4)	4.61 (3.4)	17.77 (8.5)	*H*=87.23, df=3, *P*<**0.001**
Motor phenotype	0.68 (0.6)	1.86 (0.7)	0.43 (0.4)	0.62 (0.5)	*H*=52.65, df=3, *P*<**0.001**
NART-R, estimated full scale IQ	112.84 (8.9)	104.29 (11.5)	103.04 (8.9)	113.41 (9.6)	*H*=44.22, df=3, *P*<**0.001**
MMSE, raw score	29.06 (1.0)	28.21 (1.4)	26.71 (1.4)	29.05 (1.1)	*H*=75.87, df=3, *P*<**0.001**
LM2, *z*-score	0.16 (0.9)	−0.63 (0.9)	−0.76 (1.0)	0.36 (0.75)	*H*=42.01, df=3, *P*<**0.001**
Trails B, *z*-score	0.37 (0.7)	−0.32 (1.4)	−1.58 (1.7)	−0.49 (1.3)	*H*=58.03, df=3, *P*<**0.001**
BDI-II, raw score	10.47 (7.1)	6.17 (6.0)	9.6 (5.9)	9.59 (6.5)	*H*=9.27, df=3, *P*=**0.023**
LEDD, mg/day	709.29 (482.7)	231.06 (258.9)	846.14 (513.4)	309.07 (260.1)	*H*=53.13, df=3, *P*<**0.001**

Abbreviations: BDI-II, Beck Depression Inventory-II (higher scores indicate more severe symptoms); LEDD, L-dopa equivalent daily dose; LM2, Logical Memory II; MMSE, Mini-Mental State Examination; NART-R, revised National Adult Reading Test; NTD, non-tremor dominant; RDP, rapid disease progression; TD, tremor dominant; Trails B, Trail Making Test B; YO, younger age of disease onset.

Significant values are represented in bold. Values are mean (s.d.).

**Table 2:** Mean (s.d.) for variables used to validate the four-cluster solution

	*YO (*N*=93)*	*TD (*N*=24)*	*NTD (*N*=48)*	*RDP (*N*=44)*	*Between-group differences*
Age, years	63.22 (8.6)	65.79 (8.8)	68.93 (8.1)	72.01 (7.3)	*F*=12.97, df=3, *P*<**0.001**
Disease duration	6.59 (4.7)	3.51 (2.8)	9.07 (5.7)	2.14 (1.1)	*H*=71.02, df=3, *P*<**0.001**
Hoehn and Yahr stage, raw score	1.96 (0.5)	1.54 (0.5)	2.17 (0.6)	1.99 (0.6)	*H*=18.39, df=3, *P*<**0.001**
PDQ-39 cognitive impairment dimension (Q 30–33), scaled score	22.78 (17.23)	22.57 (18.46)	33.59 (21.21)	20.39 (15.31)	*H*=13.03, df=3, *P*=**0.003**

Abbreviations: NTD, non-tremor dominant; PDQ-39, Parkinson’s Disease Questionnaire; RDP, rapid disease progression; TD, tremor dominant; YO, younger age of disease onset.

Significant values are represented in bold.

**Table 3:** Frequencies of PD-MCI and other clinical features in the four-cluster solution

	*YO (N=93), %*	*TD (N=24), %*	*NTD (N=48), %*	*RDP (N=44), %*
PD-MCI	24	42	54	25
FOG-Q item 3	41	8	69	25
SCOPA-PC item 1 and revised MDS-UPDRS item 2	11	4	23	7
SCOPA-S (NS)	19	17	17	18
SCOPA-S (DS)	24	25	40	16
ESS	22	17	46	25
REMSBD	41	29	56	32

Abbreviations: ESS, Epworth Sleepiness Scale; FOG-Q, Freezing of Gait Questionnaire; MDS-UPDRS, Movement Disorder Society Task Force Unified Parkinson’s Disease Rating Scale; NTD, non-tremor dominant; PD-MCI, mild cognitive impairment in Parkinson’s disease; RDP, rapid disease progression; REMSBD, REM Sleep Behavior Disorder Screening Questionnaire; SCOPA-PC, Scales for Outcomes in Parkinson’s disease-Psychiatric Complications; SCOPA-S (NS), Scales for Outcomes in PD-Sleep Scale night-time sleepiness subscale; SCOPA-S (DS), Scales for Outcomes in PD-Sleep Scale daytime sleepiness subscale; TD, tremor dominant; YO, younger age of disease onset.
